# Stapled hemorrhoidopexy, an innovative surgical procedure for hemorrhoidal prolapse: cost-utility analysis

**DOI:** 10.3325/cmj.2011.52.497

**Published:** 2011-08

**Authors:** Goran Ribarić, Justus Kofler, David G. Jayne

**Affiliations:** 1 European Surgical Institute, Ethicon Endo-Surgery (Europe) GmbH, Johnson&Johnson, Norderstedt, Germany; 2 St. James’s University Hospital, Leeds, United Kingdom

## Abstract

**Aim:**

To undertake full economic evaluation of stapled hemorrhoidopexy (PPH) to establish its cost-effectiveness and investigate whether PPH can become cost-saving compared to conventional excisional hemorrhoidectomy (CH).

**Methods:**

A cost–utility analysis in hospital and health care system (UK) was undertaken using a probabilistic, cohort-based decision tree to compare the use of PPH with CH. Sensitivity analyses allowed showing outcomes in regard to the variations in clinical practice of PPH procedure. The participants were patients undergoing initial surgical treatment of third and fourth degree hemorrhoids within a 1-year time-horizon. Data on clinical effectiveness were obtained from a systematic review of the literature. Main outcome measures were the cost per procedure at the hospital level, total direct costs from the health care system perspective, quality adjusted life years (QALY) gained and incremental cost per QALY gained.

**Results:**

A decrease in operating theater time and hospital stay associated with PPH led to a cost saving compared to CH of GBP 27 (US $43.11, €30.50) per procedure at the hospital level and to an incremental cost of GBP 33 (US $52.68, €37.29) after one year from the societal perspective. Calculation of QALYs induced an incremental QALY of 0.0076 and showed an incremental cost-effective ratio (ICER) of GBP 4316 (US $6890.47, €4878.37). Taking into consideration recent literature on clinical outcomes, PPH becomes cost saving compared to CH for the health care system.

**Conclusions:**

PPH is a cost-effective procedure with an ICER of GBP 4136 and it seems that an innovative surgical procedure could be cost saving in routine clinical practice.

The delivery of health care, particularly within the realms of new innovative surgical technologies, remains somewhat haphazard and subject to individual and local influences (1). This article looks at an innovative surgical procedure called stapled hemorrhoidopexy (Procedure for Prolapse and Hemorrhoids, PPH, Ethicon Endo-Surgery, Cincinnati, OH, USA) as an example of a new surgical intervention, its cost- effectiveness, and the potential influences that impact on its availability and affordability.

As previously reported, hemorrhoidal disease is a very common problem in most European populations, and in the United Kingdom (UK) has been variously estimated to affect between 4.4% to 36.4% of adults, accounting for 1% of medical consultations (2-5). Of those with symptomatic prolapsing hemorrhoids, some 10%-20% will undergo surgical intervention (6,7). The problem that hemorrhoidal disease presents to society is therefore not insignificant, and given that excisional hemorrhoidectomy (CH) is a routine, minor anorectal procedure that has been extensively studied, one might reasonably have expected a consensus view on the best modality of treatment.

The problem lies in the multitude of surgical treatments available. This includes a variety of topical agents and life-style modifications, out-patient procedures, and excisional surgery. Generally, excisional surgery is reserved for the more severe (grades III and IV) degrees of hemorrhoidal prolapse (8,9). Traditionally, in European countries the preferred CH technique was the Milligan-Morgan procedure (10). Although effective in treating hemorrhoidal prolapse, Milligan-Morgan hemorrhoidectomy is associated with significant postoperative pain often requiring a prolonged hospital admission and protracted recovery (7). For this reason, in the last decade a novel surgical technique of stapled hemorrhoidopexy has been proposed.

PPH is an innovative surgical technique that uses a specific circular stapling device (Model PPH 03 and PPH 01, Ethicon Endo-Surgery, Cincinnati, OH, USA) to reduce the hemorrhoidal prolapse and excise a band of anorectal mucosa above the dentate line. Excessive prolapsing tissue is excised and residual hemorrhoidal tissue is fixed to the underlying muscle. PPH as an innovation addresses the problem of hemorrhoidal prolapse form both an anatomical and physiological perspective, combining excision of the prolapse with resuspension (-pexy) and avoiding the painful anodermal wounds associated with other excisional procedures. Since its inception, it has been the subject of intense scientific scrutiny. Several randomized trials and meta-analyses have now shown it to be associated with significant patients benefits such as shorter operative times, less postoperative pain, reduced hospital stay, and quicker return to normal function when compared with standard Milligan-Morgan hemorrhoidectomy (11-22). Recently, in 2007, the UK National Institute for Health and Clinical Excellence (NICE) issued its updated guidance on PPH (7) and in the appraisal process it considered two economic models of cost-effectiveness, one based on an independent analysis commissioned from the UK National Health Service (NHS) Centre for Reviews and Dissemination and Centre for Health Economics in York, UK (16) and the other submitted by the manufacturer of the PPH device, Ethicon Endo-Surgery (Europe) GmbH. In both models, the differences in cost and utilities between PPH and CH were small, and therefore the incremental cost-effective ratios (ICER) were sensitive to minor changes in the assumptions made about costs and benefits. The NICE appraisal Committee noted that in both economic models the main influence on the ICERs was the utility estimates used. However, the Committee was persuaded that a clear utility benefit in favor of PPH was likely to exist, particularly in the early postoperative period.

Despite the apparent scientific support in favor of PPH, only 2285 (10%) of the 23 000 hemorrhoidectomy procedures performed in the UK in 2008-9 were done by PPH (7). In other European countries, there seems to be the same situation. This article presents a full economic analysis of PPH taking into consideration recent literature on clinical outcomes and discusses the potential influences which may contribute to the disparity observed between current best available scientific evidence and the availability of the PPH procedure in clinical practice.

## Methods

### Study design

A cohort-based model with a decision tree structure was developed to investigate the cost utility of PPH compared with CH. The model is run for two cohorts of patients with one group receiving a PPH on initiation and the other a CH.

The patient then goes through a recovery period during which a proportion of the patients may suffer a recurrent prolapse. The severity of the recurrent prolapse determines whether the patient is able to self-treat or requires further surgery. For this reason, the patients could follow one of four pathways through the model:

 1) full recovery with no recurrent prolapse;

 2) severe recurrent prolapse requiring re-operation followed by no further prolapse;

 3) severe recurrent prolapse requiring re-operation followed by a second recurrent prolapse;

 4) less severe recurrent prolapse which can be self treated.

The following economic parameters were included as the primary outcomes of the cost- effectiveness analysis: cost per procedure at the hospital level, total direct costs from the perspective of UK NHS, and Quality Adjusted Life Years (QALY).

The time-horizon was set at 1 year, because it has been suggested that there is no difference in treatment effects after 1 year and that any prolapse beyond that time could be seen as de novo hemorrhoidal prolapse, rather than disease recurrence due to treatment failure (12).

### Data inputs

*Effectiveness data and valuing health benefits.* A systematic review of the current best available clinical evidence to evaluate the short- and long-term clinical outcomes of PPH compared with CH for symptomatic prolapsing hemorrhoids in people of any age has been recently published by authors of this article (17) and is used as reference for cost-effectiveness data in this economic evaluation. In total, 34 randomized controlled trials (RCT) and two systematic reviews were identified that compared PPH with CH. Three studies comparing other techniques than Milligan-Morgan/Ferguson and 2 studies with poor quality or not available in English language were excluded. Therefore, 29 RCTs were included in the systematic review. Data were extracted independently for each study and differences were analyzed with fixed and random effects models (17). The main clinical outcomes for the cost-effectiveness analysis were recurrent prolapse rate, operation time, and length of hospital stay.

Results from the meta-analysis of the systematic review (17) show that operation time for PPH was significantly shorter (weighted mean difference – 13.79 minutes) than for Milligan-Morgan hemorrhoidectomy. The percentage of patients who experienced a re-operation because of recurrent prolapse was 27.2% and 66.2% for CH and PPH, respectively (16).

Based on the NICE, UK reference case, the relative treatment effect of PPH compared to CH in terms of QALYs was estimated using a generic measure of health related quality of life (HRQoL). QALYs are calculated by multiplying the length of time in a particular health state by its corresponding utility value. Since data were not estimated directly in any trial, they were estimated indirectly by synthesizing evidence from a number of sources (7,16).

### Costing

For cost calculation, the different resource use was combined with the corresponding costs for the PPH or CH procedure. For the total surgery costs, time spent in operation theater was estimated and was combined with the cost per minute of surgery and the cost of the stapling device. The price of the hemorrhoidal circular stapler (PPH03, Ethicon Endo Surgery) was set at the list price of GBP 440 (US $ 702.46, €497.02) for the year 2009. The cost of hemorrhoidal surgery (GBP 8.27 per minute, US $13.20, €9.34) in the operation theater was based on the published assessment report (16) for NICE appraisal TA 128. Cost of hospital stay amounted to be GBP 282 (US $450.21, €318.49) per day determined from the NHS reference costs 2007-2008 (Excess Bed Day, FZ22A) (23).

In order to include the cost of recurrent hemorrhoidal prolapse within a year from the NHS perspective, the probability of re-surgery for a patient who experienced a recurrent prolapse was combined with the total hospital costs of the repeated procedure.

### Sensitivity analyses

One-way sensitivity analysis was performed to test the robustness of the results. The ICER is the ratio between the difference in costs and the difference in utility (QALYs) of the two interventions.

Therefore, the impact on the ICER was explored by the variation of theater and bed day costs (%).

Furthermore, it is highly likely that the hemorrhoidal re-prolapse rate and hence the re-surgery rate for PPH may be lower than the 10.1% and 66% used in the base case model. It is very well known that these figures are influenced by surgical experience and the volume of procedures undertaken. For example, the long-term outcomes of a multicenter randomized clinical trial with 100 patients has demonstrated that the re-surgery rate for PPH can be even 50% lower than assumed in base case model (33% instead of 66%), whereas the re-surgery rate for CH remains roughly the same (25%) (24). Based on this conservative approach, a sensitivity analysis was conducted to determine how the ICER was affected when either or both the recurrence rate of prolapse and the re-surgery rate were changed.

## Results

### Baseline analyses

The cost calculation results of total hospital costs for each procedure are shown in Table 1 and amount to a total of GBP 996 (US $1590.11, €1125.18) and GBP 1022 (US $1631.62, €1154.45) for PPH and CH, respectively. The decrease in time in the operating theater and shorter hospital stay with PPH led to a cost-saving of GBP 27 (US $43.11, €30.50) on the hospital level compared with CH for each procedure. From the NHS perspective, the total treatment costs, inclusive of the cost of recurrent prolapse, led to an incremental cost of GBP 33 (US $52.68, €37.29) after one year (Table 2).

**Table 1:** Total hospital cost of conventional excisional hemorrhoidectomy (CH) compared to stapled hemorrhoidopexy (PPH)

	Resource use		Cost unit (GBP*)	Total costs (GBP)		Difference (GBP)
Parameter	CH	PPH		CH	PPH	
Theater (min)	31.20	17.41	8.27	258	144	-114
Hospital stay (day)	2.71	1.46	282	764	412	-353
Staple gun (unit)	0	1	440	0	440	440
Total hospital cost				1022	996	-27

*GBP 1 = US $1.60, €1.12.

**Table 2:** Treatment cost in one year of conventional excisional hemorrhoidectomy (CH) compared to stapled hemorrhoidopexy (PPH) from the health care societal perspective

	Probability (%)		Total costs (GBP*)		Difference (GBP)
Parameter	CH	PPH	CH	PPH	
Initial surgery			1022	996	-27
Recurrent prolapse	2.60	10.10			
Resurgery for prolapse	27.20	66.20	7	67	
Treatment cost (one year)			1029	1062	33

*GBP 1 = US $1.60, €1.12.

QALYs were estimated as shown in Table 3 and induced an incremental QALY of 0.0076 (7). Improvements in HRQoL and decrease in post operative pain and complications with PPH led to greater total QALYs vs CH (16) and showed an ICER of GBP 4316 (US $6890.47, €4878.37), which is a highly cost-effective result.

**Table 3:** Incremental cost-effectiveness ratio (ICER) of stapled hemorrhoidopexy (PPH) compared to conventional excisional hemorrhoidectomy (CH) for the treatment of hemorrhoids

Procedure	Cost (GBP*, mean)	QALYs (mean)^†^	ICER (GBP)
PPH	1062	0.77	4316
CH	1029	0.76	
Difference	33	0.0076	

*GBP 1 = US $1.60, €1.12.

†QALY – quality adjusted life years.

### Sensitivity analyses

**Figure 1:**
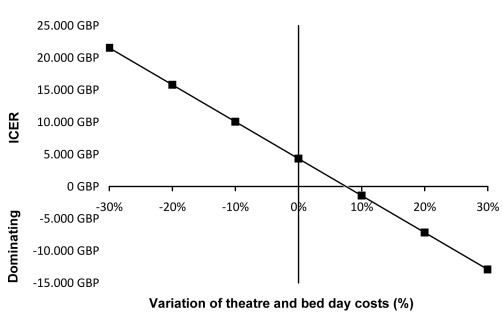
Incremental cost-effectiveness ratio (ICER) sensitivity analysis for stapled hemorrhoidopexy compared to conventional excisional hemorrhoidectomy exploring impact of theater and bed day costs (%). GBP 1 = US $1.60, €1.12.

One-way sensitivity analyses for several parameters indicated that the results were robust. Figure 1 shows the variation of ICER for PPH compared to CH when the operation theater costs and hospital stay costs are up to 30% higher or lower than in the base case. Even if the hospital cost were 30% lower, PPH would still be cost-effective with an ICER of GBP 21 551 (US $34 406.07, €24 354.78) from NHS perspective.

**Figure 2:**
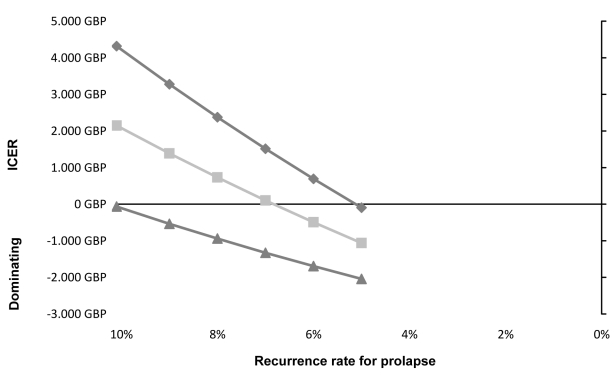
Sensitivity analysis for stapled hemorrhoidopexy showing incremental cost-effectiveness ratio (ICER) as a function of recurrence rate of prolapse and depending on a change of re-surgery rate for stapled hemorrhoidopexy (re-surgery rate for excisional hemorrhoidectomy remaining at 25%). Rhombs – re-surgery base case; squares – re-surgery -25%; triangles – re-surgery -50%. GBP 1 = US $1.60, €1.12.

A sensitivity analysis was conducted to determine how the ICER changed with a postoperatively lower hemorrhoidal re-prolapse rate than calculated in the base case and showed that the PPH can become cost-saving compared with CH procedure also for the national health care system (Figure 2).

## Discussion

In the cost-utility analysis of PPH presented in this article, a decrease in operating theater time and shorter hospital stay associated with PPH led to a cost-saving compared to CH of GBP 27 per procedure at the hospital level and to an incremental cost of GBP 33 after one year from the societal perspective. Calculation of QALYs induced an incremental QALY of 0.0076 and showed an ICER of GBP 4316.

It should be emphasized that in the economic model base case presented in this article, the recurrence rate of re-prolapse was set at 10.1% for PPH and it was assumed that 2/3 or 66% of patients out of the re-prolapse group would require surgical re-intervention (16). Taking into consideration the published evidence (24) that suggest that re-surgery rate for hemorrhoidal prolapse can be even 50% lower than assumed in conservative approach of the base case model (33% instead assumed 66%), the PPH procedure becomes cost-saving compared to CH also for the national health care system.

Although the literature on PPH is quite extensive, only a few studies have included a cost-analysis (19,24-28). In one randomized controlled trial, the costs in one hospital were measured and the authors concluded that CH was more expensive than PPH, with the cost of the stapling device being more than offset by the decreased operating time and shorter hospital stay (19).

The previously published cost-analysis studies all have their limitations. Often the costs are not presented in enough detail and are partly taken from a single hospital. There is frequently not enough transparency in how the costs were derived and the methodology is poorly described. Furthermore, costs are often only considered at the hospital level and not widened to the societal level. In only one study were costs analyzed from the perspective of a health care provider, but the study used cost input from 1998 and the authors presented no outcomes of cost-effectiveness (27). The first full economic evaluation assessing cost-effectiveness was only undertaken at the time of the NICE, UK health technology assessment in 2007, which concluded that PPH was clinically effective, offering immediate benefits in terms of reduced postoperative pain and faster return to normal activities, and that consequently PPH seems to be a cost-effective surgical procedure (7). However, well known differences in surgical clinical practice where different reccurent prolapse rates might be detected by different surgeons were not considered.

The full economic evaluation of PPH in this article shows that one of the major drivers of cost-effectiveness of PPH may be the total hemorrhoidal re-prolapse as well as the total re-intervention rate following PPH and CH.

As previously published in systematic reviews of randomized trials, contrary to the hemorrhoidal re-prolapse rate, the difference between PPH and CH regarding the total re-intervention rates did not reach significant difference either for non-surgical re-interventions or surgical re-interventions (7,17,22) and the rate of hospital readmissions after PPH compared to CH was reported to be without significant difference.

The only significant difference between PPH and CH in the postoperative period was reported to be the rate of hemorrhoidal re-prolapse and consequently the rate of re-surgery regarding the re-prolapse, as a prominent subgroup of total surgical re-intervention rates. Because of the conservative nature of the model, the difference in re-surgery rate for re-prolapse was used for calculation instead of the total re-intervention rate, which has been used by others.

This article expands on previous cost-effectiveness analysis, taking into consideration the differences in recurrent prolapse reported between PPH and CH. In spite of a reported 4-fold increase in hemorrhoidal re-prolapse rate following stapled hemorrhoidopexy, the PPH procedure remains highly cost-effective and can even lead to cost savings. The explanation is likely based on the evidence that about 50% of patients with the hemorrhoidal re-prolapse postoperatively do not seek further surgical intervention and are quite satisfied with the surgical results (17). Also, it appears to be particularly true if surgeon experience and surgical volume increase with a concomitant decrease in the re-prolapse and re-intervention rates.

A limitation of the presented cost utility analysis is that the economic model is confined to a one year time horizon. This however is not considered a significant limitation as there appears to be convergence in utilities by one year. Clinical opinion also considers that prolapse beyond this point is a new prolapse, not necessarily a recurrence of previously prolapsing hemorrhoids. Furthermore, the model did not consider complications and symptoms, other than prolapse. However, the difference between PPH and CH regarding the total re-intervention rates did not reach significant difference either for non-surgical re-interventions or surgical re-interventions (7,17,22).

If seen in a broader health care context, given the positive recommendations of the 2007 NICE, UK guidance and its obvious international impact on the European network for Health Technology Assessment (EUnetHTA), it is therefore somewhat surprising that disparities still exist within the European health care systems between the availability of an innovative surgical procedure such as PPH and current best available scientific evidence.

In terms of local health care policy and delivery, managers are frequently dissuaded by the cost of new surgical technologies in comparison to traditional techniques. The manager with targets and financial budgets to meet is likely to be less concerned by any societal benefits that a new procedure may offer than the immediate costs to his institution. Unless managers are unaware or choose to ignore international NICE guidance, this should not influence the provision of stapled hemorrhoidopexy.

From a clinical perspective, of the three possible processes which might underlie the variation in surgical practice (patient referral patterns, choice of surgical intervention, and health care delivery), it therefore appears that it is the clinician’s preference that remains the predominant factor. It is hoped that the economic evaluation presented in this study, in line with previous NICE guidance, will reassure clinicians that stapled hamorrhoidopexy performed with PPH device is a cost-effective treatment, and even when recurrent prolapse is taken into consideration, it has the potential to be a cost-saving surgical procedure. Further education and training in the proper technique of PPH should eliminate any remaining unfamiliarity and help to eliminate the current disparity between health care provision and current best available evidence.

PPH seems to be an attractive alternative to CH in patients with symptomatic, prolapsing hemorrhoids. Offering obvious clinical advantages, including less postoperative pain, reduced hospital stay, and quicker return to normal function, PPH presents a cost-effective procedure from the societal perspective with an ICER of GBP 4316. It seems that an innovative surgical procedure such as PPH could be even cost-saving in routine clinical practice.

To ensure uniformity in health care provision, the variation in individual surgical practice needs to be addressed, and clinicians and managers made aware of the economic arguments in favor of stapled hemorrhoidopexy.

In the authors’ opinion, any residual doubts relating to potential cost-savings and unfamiliarity with an innovative surgical procedure such as stapled hemorrhoidopexy can be overcome with appropriate and continuous surgical training combined with the rigorous implementation of evidence-based medicine in health care decision making.
